# Supplementary material for: Polyketide synthase gene clusters in the ericoid mycorrhizal fungus Oidiodendron maius: genome-wide prediction, expression profiling, and functional characterization of knockout mutants
Source: Front Plant Sci. 2026 Sep 16;17:1924430. doi: 10.3389/fpls.2026.1924430 (PMC13623903; doi:10.3389/fpls.2026.1924430)
Supplement: Supplementary file 1 [file SupplementaryFile1.zip › Supplementary Materials/Supplementary Folder 1.zip!/Oidma1_AssemblyScaffolds_Repeatmasked/knownclusterblast/cluster16/jgi.p_Oidma1_120065_mibig_hits.html]

| MIBiG Protein | Description | MIBiG Cluster | MiBiG Product | % ID | % Coverage | BLAST Score | E-value |
| --- | --- | --- | --- | --- | --- | --- | --- |
| BAV19380.1 | NRPS-like\_enzyme | BGC0001390 | LL-Z1272beta\_biosynthetic\_gene\_cluster | 28 | 98.6434108527 | 277 | 8.4e-74 |
| AAS98174.1 | ochratoxin\_A\_non-ribosomal\_peptide\_synthetase | BGC0001030 | Ochratoxin\_A\_biosynthetic\_gene\_cluster | 26 | 63.1782945736 | 203 | 1.2e-51 |
| EAA65602.1 | hypothetical\_protein | BGC0000022 | Asperfuranone\_biosynthetic\_gene\_cluster | 29 | 39.7286821705 | 112 | 3.6e-24 |
| ABW71853.1 | nonribosomal\_peptide\_synthetase | BGC0000303 | Anthramycin\_biosynthetic\_gene\_cluster | 27 | 30.0387596899 | 74 | 6.3e-13 |
| AEA29644.1 | putative\_nonribosomal\_peptide\_synthetase\_and\_kinurenine\_monooxygenase | BGC0000409 | Porothramycin\_biosynthetic\_gene\_cluster | 25 | 40.988372093 | 73 | 1.8e-12 |
| AEC14349.1 | nonribosomal\_peptide\_synthetase | BGC0000377 | Koranimine\_biosynthetic\_gene\_cluster | 22 | 33.5271317829 | 70 | 1.6e-11 |
| AAG31130.1 | MxcG | BGC0001345 | Myxochelin\_biosynthetic\_gene\_cluster | 26 | 30.523255814 | 69 | 3.5e-11 |
| XP\_001220460.1 | hypothetical\_protein | BGC0001182 | Chaetoglobosins\_biosynthetic\_gene\_cluster | 26 | 30.4263565891 | 60 | 1.2e-08 |
| ACS68554.1 | hybrid\_PKS-NRPS\_protein | BGC0001026 | NG-391\_biosynthetic\_gene\_cluster | 23 | 41.3759689922 | 56 | 1.8e-07 |
